# Exploring emerging learning needs: a UK-wide consultation on environmental sustainability learning objectives for medical education

**DOI:** 10.5116/ijme.5643.62cd

**Published:** 2015-12-24

**Authors:** Sarah C. Walpole, Frances Mortimer, Alice Inman, Isobel Braithwaite, Trevor Thompson

**Affiliations:** 1 Hull York Medical School, York, UK; 2 Centre for Sustainable Healthcare, Oxford, UK; 3 Medical School, University College London, UK; 4 Medical School, University of Bristol, UK

**Keywords:** Curriculum development, professionalism, education research, social determinants of health, environmental sustainability

## Abstract

**Objectives:**

This study aimed to engage wide-ranging stakeholders and develop consensus learning objectives for undergraduate and postgraduate medical education.

**Methods:**

A UK-wide consultation garnered opinions of healthcare students, healthcare educators and other key stakeholders about environmental sustainability in medical education. The policy Delphi approach informed this study. Draft learning objectives were revised iteratively during three rounds of consultation: online questionnaire or telephone interview, face-to-face seminar and email consultation.

**Results:**

Twelve draft learning objectives were developed based on review of relevant literature. In round one, 64 participants’ median ratings of the learning objectives were 3.5 for relevance and 3.0 for feasibility on a Likert scale of one to four. Revisions were proposed, e.g. to highlight relevance to public health and professionalism. Thirty three participants attended round two. Conflicting opinions were explored. Added content areas included health benefits of sustainable behaviours. To enhance usability, restructuring provided three overarching learning objectives, each with subsidiary points. All participants from rounds one and two were contacted in round three, and no further edits were required.

**Conclusions:**

This is the first attempt to define consensus learning objectives for medical students about environmental sustainability. Allowing a wide range of stakeholders to comment on multiple iterations of the document stimulated their engagement with the issues raised and ownership of the resulting learning objectives.

## Introduction

Environmental change is among the greatest challenges to health and healthcare of our time. Organisations including the World Health Organisation are emphasising the centrality of working towards environmental sustainability to protect public health.^1^ Environmental change significantly impacts the diseases that health professionals see and treat. Moreover, environmentally sustainable practices offer great opportunities to improve public health and healthcare services. Sustainable development –“*development that meets the needs of the present without compromising the ability of future generations to meet their own needs*”^2^ – offers many health‘co-benefits’.^3^ Health services have a large ecological footprint. Reducing this footprint, saving money and improving patient care can go hand in hand.^4^

Medical education must prepare medical students to be professionals in, and leaders of, health systems. It must equip students with the knowledge, skills and attitudes to provide environmentally sustainable services. According to the World Medical Association, medical professionals should understand how environmental change affects the health of individuals, communities and societies.^5^ The translation of emerging health topics into medical curricula may begin ‘bottom-up’, with individual educators incorporating new material locally; or ‘top-down’, guided by evidence or recommendations from standard-setting or regulatory bodies. To date, introduction of teaching about environmental sustainability has generally been ‘bottom-up’, without a conceptual framework or expert recommendations.^6^ Such ‘bottom up’ approaches range from action research projects with goals defined by learners (e.g. with nurses in USA^7^ and in Brazil^8^), to introduction of seminar-based, lecture-based or online teaching to address sustainability topics (e.g. through BMJ online^9^). While the bottom up approaches may bring smaller scale change, working ‘top down’ to impose new requirements may bring only superficial change; an ideal is to combine both approaches.^10^ Hence the need to develop learning objectives to bring authority and uniformity to guide curricular change from above, and to complement the engagement and learning achieved through ‘bottom up’ implementation of teaching to date.

This consultation was carried out at the request of the UK’s General Medical Council (GMC) and aimed to identify learning and teaching needs related to the topic of environmental sustainability. Various methods have been used to develop guidelines about emerging topics in medical curricula; many draw on the policy Delphi method.^11^^-^^14^ Others have not done so explicitly, but have also sought to build consensus amongst stakeholders through consultation and iterative methodology.^15^^,^^16^ A modified Delphi explored learning objectives for a course about environment and sustainability, and, like this study, involved a wider pool of respondents (n=188) in round one and a smaller group in round two to further explore ideas and develop recommendations.^17^

The primary aim of this study was to address a lack of agreement on required learning about environmental sustainability, and create a consensus on learning objectives that should be included in undergraduate and postgraduate medical education (primarily in the UK, but potentially with transferability to other settings). Secondary aims were to engage a wide-range of stakeholders in medical education, and elucidate their views on the learning needs related to environmental sustainability of medical students and postgraduate doctors.

## Methods

This consultation took the format of a policy Delphi, modified to involve a large number and range of stakeholders. The policy Delphi structures communication to allow a range of stakeholders to deal with a complex problem. The process seeks to build consensus by identifying divergent opinions, rather than make decisions on the basis of simple votes.^18^ The process is designed to guide policy analysis by structuring feedback from stakeholders who may have conflicting views, to ensure that all options and opinions are considered and the impact and acceptability of any decision is estimated.

The policy Delphi approach is appropriate for research in medical education because it recognises that actors with varying levels of knowledge and understanding of the topic area have valuable contributions to make, ^19^ and the definition of ‘expert’ can extend to individuals who have experience of acting or advocating about the issue, have researched and informed themselves about the issue, and/or have an understanding of related or overarching concepts. Another benefit is that the policy Delphi method facilitates participants to learn more about the subject during the process of building consensus.^20^

Central to the policy Delphi are structured rounds of feedback, anonymity for participants, and opportunity to revise opinions based on interaction with other participants. Qualitative information is utilised by the committee to formulate the required policy, with inductive development of the consultation in response to feedback at each stage.

This policy Delphi is embedded in a constructivist paradigm, which falls within the interpretive paradigm. The constructivist paradigm is appropriate for this research question; one which cannot be answered through precise analysis of data and instead requires collection and integration of subjective judgments.^21^ In designing this study, we aimed to collect qualitative data to generate theories about the required learning in the sustainability domain. We aimed to assimilate and integrate the variety of subjective contributions through a process that allowed participants to develop their own understanding and contribute and agree to a consensus document. Whilst a challenge of using the Delphi approach is to ensure transparency and to recognise the influence of the committee who initially set the agenda, and in applying the findings from each round of consultation, a strength of the Delphi over other techniques such as brainstorming is that it provides all participants with a more equal voice rather than only the strongest opinions or loudest voices dominating the discussion.^21^

The committee for this consultation comprised four healthcare educators and two students, all with knowledge and teaching experience in the environmental sustainability domain. The first round enabled many stakeholders to comment on draft learning objectives drawn up by the committee. Round two involved an interactive seminar which allowed participants to understand, discuss and modify the learning objectives further. In round three, all participants were invited to comment on the revised learning outcomes and validate that their contributions were reflected.

Modification of the policy Delphi for this consultation involved draft competencies being developed by the authors with the aim of stimulating responses in round one, as it was felt that (in comparison with more open-ended questions) this would prompt participants to give more specific responses about the range of topics covered, the level of depth, and any particular areas they felt were strong or weak. Development of the initial draft learning objectives was informed by key papers on environmental sustainability and health^22^^-^^24^ and existing teaching on environmental sustainability in UK medical education^25^ (Appendix 1). Use of a background document allowed a wider number of participants (including those with limited prior engagement with environmental issues) to participate in the consultation, which was important because of the breadth of the topic area and range of disciplines and approaches with which it intersects. Including a wider range of participants in the development of the consensus is also important for this Delphi because there is no evident community or group of experts for this rapidly, but relatively newly, emerging area of medical education. The larger number of participants increases the possibility of incorporating a wider range of viewpoints.

In round one, an online questionnaire asked participants to rate the perceived importance and feasibility of implementing each learning objective; the coherence, relevance, format and feasibility of the whole document; and the importance and feasibility of individual proposed learning objectives, in each case on a four-point Likert scale. It also solicited suggestions for improvement. Invitations to participate were sent to 88 medical education and leadership institutions to cascade to others in their organisations. The heads of all 33 UK medical schools, all UK postgraduate deaneries and all medical Royal Colleges were invited to complete the questionnaire online or via telephone interview. The questionnaire remained open for 13 weeks. Thematic analysis of feedback identified suggestions for additions and alterations to content and structure, including comments that were reciprocal or refutational. Wherever possible, suggested alterations were incorporated.

In round two, a face-to-face seminar in London, the revised draft was shared with participants. All UK medical schools and the institutions contacted in round one were invited to send a representative member of staff. Members, including medical students, of the Sustainable Healthcare Education network were also invited to attend. Participants were all those who responded positively to this invitation. During this consultation, a World café; format^26^ was used in which delegates could spend time at three tables, which each had a different theme drawn from the contested issues in round one. These discussions helped to explore tensions which had arisen during round one. The facilitator at each table documented comments by taking notes during the discussion. Participants left anonymous written feedback at the end of the seminar. All comments and feedback were transcribed and organised into three pre-defined categories: (a) content of learning objectives, (b) structure and presentation of learning objectives, or (c) methods for implementation. Through discussion to reach consensus between the committee, all (a) and (b) responses were reviewed and where possible incorporated into a third draft of the learning objectives. Refutational responses were given particular consideration.

Round three aimed to ensure that participants’ comments were interpreted correctly and had been incorporated as far as possible, and that participants could agree with the final learning objectives, whilst acknowledging that it is not possible to fully resolve all differences of opinion. The third draft learning objectives were circulated to all round one and two participants, with an invitation to comment. All comments were taken into account in a final refinement of the learning outcomes document.

All data were anonymised. All participants were informed of the study’s nature and format on entry into the consultation, and could withdraw from the study at any time.

## Results

### Participants, sample size and sampling methods

There were 64 individual responses in round one (Table 1). Not every respondent stated which institution they are from, but the institutions that were represented included 25 universities, 6 postgraduate deaneries, 6 hospitals, one Royal College and four health agencies or NHS bodies. Some group responses were also received: two written submissions representing views collated in an organisation, and three reports of focus groups within different UK medical schools. 

**Table 1:** Backgrounds of round one participants

Participants	n
Medical Educators – undergraduate	20
Medical Educators – postgraduate	15
Students – medical	12
Trainee doctors	5
Health service managers	2
Educators – postgraduate, non-health	1
Sustainability experts	1
Students – allied health	1
Other – allied health	7

In round two, 33 invitees attended the seminar, including healthcare leaders from the UK’s General Medical Council, Department of Health, Royal Colleges and NHS (n=6); undergraduate medical educators from eleven medical schools (n=13) and five postgraduate deaneries (n=8); medical students from five medical schools (n=6).

In round three, 88 participants were contacted and to reply if they opposed any of the learning objectives or wanted to suggest any further edits. Acknowledgement, endorsement and suggestions for edits were received from seven participants, but the suggestions for edits were seen by the committee to contradict findings from previous rounds or go beyond the scope of the document. When this was suggested to the respondents, they agreed that the edits should not be made.

### Data collection and analysis, Round one

In round one, median (and mean) ratings of the whole document were: coherence 4.0 (3.4), relevance 3.5 (3.3), formatting 3.0 (3.3) and feasibility 3.0 (2.7). In all areas, ratings ranged from one to four. Of the ten learning objectives, two had median feasibility rating of four, seven had median feasibility rating of three and one had median feasibility rating of 2. Importance ratings were higher, with seven having a median importance rating of 4.

Qualitative feedback from round one was overall positive, commenting that the learning objectives were both clear and applicable:

“It is logical, evidence based and mapped to the curriculum.” Medical educator - postgraduate

“Clear narrative that moves through ‘why’ it is an issue, ‘what’ can be done and ‘how’ progress can be measured and achieved.” Health service manager

Concerns and suggestions for improvement of the learning objectives emerged under four main themes: specific content, relevance and scope for application to medical curricula, feasibility of implementation, and format and presentation; Table 2 shows illustrative subsidiary themes.

**Table 2:** Themes emerging from analysis of qualitative data from phase one

Heading – main theme	Subthemes (examples)
Specific content – proposed additions	Clear definition of sustainability
	Social and economic dimensions of sustainability
	Population growth/ control
	Nutrition
	Sustainable/ ethical procurement
	Sustainable healthcare research
	Health service resilience
	Waste management
	Conflicting priorities, e.g. infection control by introducing single use equipment versus preserving and reusing resources
Relevance and scope for application to medical curricula to medical education	Relevance to health and healthcare not always explicit
	Undergraduate versus postgraduate
	Core versus optional
	Stand-alone versus integrated
Feasibility of implementation	Too many learning objectives, too wide-ranging
	Too prescriptive (rather than encouraging critical thinking)
	Too much theory, need to be more relevant to practice
	Need to link to existing curriculum topics
	Need for training and CPD for educators
Format and presentation	Objectives and format should align more closely with the GMC’s ‘Tomorrow’s Doctors’
	Avoid jargon
	Need for multiple documents, some simpler

### Specific content

Several participants advised that central concepts, such as sustainability, should be defined. Most comments supported the learning objective describing the relationship between environment and human health, e.g. “*essential for all professionals including doctors*” (Medical educator- Postgraduate) and “*vital background, much needed*” (Trainee doctor). In contrast, one participant stated that they felt this to be “*more about being a good world citizen than a good doctor*” (Medical educator - postgraduate) and another that it “*should have been covered in school … [and is] basic stuff for the first medical school term*” (Medical Educator- Postgraduate).

One challenge was to balance the addition of important topics with the need for the final document to be concise, accessible and feasible to implement.

### Relevance and scope for application to medical curricula

Participants recommended more clarity on the links between the learning objectives and the practice of medicine, and advice on where and how the learning objectives should be incorporated. The committee added clarification that the objectives were primarily intended for core undergraduate curricula, but may inform non-core modules and be relevant to postgraduates.

Responses also suggested that different types of curriculum must be considered, and that supporting materials need to cater for problem-based learning as well as traditional curricula. Respondents also discussed whether the outcomes were intended for integration within specialty teaching or included as stand-alone elements.

### Feasibility of implementation

Participants highlighted space constraints in the medical curriculum and the challenge of teaching about environmental topics in enough detail to give students adequate sufficient understanding. A hierarchy of importance was suggested to assist educators in identifying key areas to incorporate.

“If there was going to be one hour of teaching what are the 1 or 2 key things you would like taught in that hour?” Vice Dean – undergraduate medical education.

“The main thing is to emphasise that most can be integrated into existing teaching. The more examples of successful practice that are included the better.” Undergraduate medical educator

### Format and presentation

Practical suggestions were made about rewording and restructuring to make the draft learning objectives more accessible, user-friendly or effective.

The original twelve proposed learning objectives were condensed into three headline objectives with more detailed supporting material, in order to highlight priority concepts and issues while still addressing other important topics.

### Data collection and analysis, Round two

Informed by the themes which arose during round one, the committee identified six areas to explore further: (1) helping educators and students to engage (2) teaching ethical aspects of environmental sustainability, (3) the impacts of long-term healthcare trends on sustainability and related teaching, (4) integrating sustainability across the curriculum, (5) implementation and teaching delivery, and (6) improving wording and structure (Appendix 2). Areas two and three relate to the content of the learning objectives.

On the subject of ethics, participants debated where environmental issues fall in relation to the duties of citizens and the duties of doctors. They discussed whether it is the role of medical education to address issues that every citizen should address, whether or not they are a health professional. Other topics of discussion were the relevance or otherwise of the Hippocratic Oath and the principle of non-maleficence, whether knowing and supporting the laws and policies of healthcare institutions falls within the duty of a doctor, and the extent to which advocacy about environmental issues is a doctor’s role. Participants also considered at what stage in training it is most useful to present ethical issues for trainees to explore.

On the topic of long term trends in healthcare, participants debated the extent to which doctors require an understanding about health systems and resource use. It was suggested that to enhance students understanding, they need to be able to define sustainability. It was further suggested that learning about sustainability may enhance students’ understanding of public health and social determinants of health, and help them to see the relevance of these to their clinical practice.

Some feedback could not be incorporated due to contradictory responses. For example, regarding the original learning objective about resource use, comments were:

“This area would not be considered essential for a graduate to be competent F1 doctor, perhaps something to develop as a postgraduate.” (Educator– undergraduate medical)

“Very important to understand resources and the effect of these. I think quantifying the impact is much too in depth.” (Student – medical)

Where advice was contradictory, decisions were made about which to follow based both on reference to the published literature and the expertise of the committee members. Some comments were felt to be beyond the scope of the learning outcomes, such as ethical procurement of medical equipment and respect for different cultures.

Responses related to implementation will inform future work. Key responses in this category included recognition that development of knowledge, skills and confidence of medical educators in this area is needed, and that supporting materials should be adapted to different curricula.

### Data collection and analysis, Round three

In round three, seven responses were received. None suggested edits that were perceived to be in line with comments from previous rounds or upheld by the respondent when they were questioned about this, therefore no further edits were made to the learning objectives.

The final document contains three priority learning objectives aligned with the GMC’s categories of doctor as scholar, doctor as practitioner and doctor as professional (Appendix 3). As a scholar, doctors require an understanding of how the environment and human health interact at different levels. As a practitioner, doctors must be able to apply knowledge and skills around sustainable healthcare in order to improve the environmental sustainability of health systems. As a professional, doctors must consider the ethical issues posed by the relationship between the environment and health, which was framed in terms of how the duty of a doctor to protect and promote health is shaped by the dependence of human health on the local and global environment.

## Discussion

This three-stage consultation addresses the current lack of specific guidance^6^ on what learning related to sustainability and environmental issues undergraduate and postgraduate doctors should achieve and aims to provide a framework for curriculum development to address some of the problems encountered in developing learning objectives in a ‘bottom-up’ manner, as has been done elsewhere for subjects such as quality improvement^27^ and communication skills.^15^

Participants almost unanimously supported the need for consensus learning objectives to inform teaching and curriculum development. The GMC outlines the duty of a doctor to protect and promote health,^28^and participants recognised sustainability as fundamentally linked to this duty. Through the consultation, some topics were added (e.g. health co-benefits of sustainable behaviours) or expanded (e.g. ethical challenges associated with delivering environmentally-sustainable healthcare) from the initial draft document, while its structure was simplified to facilitate implementation. To address the many dimensions of sustainability, one learning objective addresses public health dimensions, another treats practical aspects in healthcare provision, and the third encourages exploration of ethical issues.

Consensus was reached amongst participants that the document should not dictate whether the objectives should be introduced as a stand-alone curricular topic or as a perspective through which to approach existing topics (such as health inequalities, ethics and leadership), leaving such decisions to individual medical schools.

### Methods, strengths and limitations

The consensus learning outcomes are consistent with curriculum recommendations from professional bodies^29^^-^^31^and peer-reviewed literature,^32^-^34^ suggesting that the results are transferable to medical curricula. Confirmability of the study is enhanced as rigorous reporting took place throughout the study period. Such reporting was regularly referred to at the time of write up, to reflect on the process and reflexivity.

The consultation was designed to enable dialogue and overcome the difficulty of eliciting meaningful input from stakeholders on a potentially unfamiliar topic. It is suggested that the Delphi technique is useful where ethical and social issues are paramount, rather than economic or technical problems. All of these are relevant to sustainability, and our study has primarily drawn out ethical and social tensions while recognising the need for further exploration of how economic and technical developments relate to the topic of sustainability and the learning needs of future doctors.

Anonymity of written responses in all three rounds allowed participants to give their views freely,^19^ which is a strength of this study. A disadvantage of anonymous responses that has been proposed is the lack of accountability and traceability means that responses may be given that would not be supported by the participant.^36^ Participants offered wide-ranging perspectives and expertise, which is important to ensure that different societal, health service and individual needs are considered. Untutored responses are useful in developing recommendations that are acceptable to a broad audience.

The design of this study allowed the inclusion of views from a relatively large number of participants, which increases the dependability of the results and their ability to represent the views of the larger population.^37^ We acknowledge, however, that the sample size could have been larger to incorporate more views, especially of educators from around the UK. The process of exchanging views and allowing participants to modify their opinions and helped to address the complexity of this subject.

### Future research

Validation of the acceptability, feasibility and usefulness, or otherwise, of the learning objectives will primarily be in the extent to which the learning objectives are implemented. As highlighted by participants, work is needed to give educators the skills and confidence to facilitate implementation.

The methods of this consultation can inform future work to set priorities in medical education. The method allows input from a range of stakeholders, and multiple revisions, which is particularly useful where there is not a small or well-defined stakeholder group, such as with the topic of environmental sustainability. In our increasingly interdependent and globalized world, complex systems and factors affect health and healthcare, and this approach lends itself to studying such complex topics.

Support for educators and curricular leads will aid implementation of these learning objectives. Future research may investigate students’ baseline knowledge about environmental issues, effective pedagogies for learning and its impact on health professionals’ practice.

## Conclusions

This three-stage consultation has identified learning objectives on the topic of environmental sustainability for tomorrow’s doctors. The learning objectives reflect the roles of a doctor as scholar, practitioner and professional.

The consultation advances our understanding of how environmental sustainability relates to medical education, by exploring the views of medical educators, students, healthcare professionals, and representatives of medical schools and other influential health organisations. It demonstrates methods for developing a consensus document in collaboration with a wide range of stakeholders. The outcome is a simple and adaptable educational framework that can inform both teaching and curriculum design.

### Acknowledgements

We would like to thank Stefi Barna, the University of East Anglia and all participants. Funding was provided by the Higher Education Authority (grant) and Universities of Bristol and East Anglia (staff time and Cabot grant towards seminar costs).

### Conflict of Interest

The authors declare that they have no conflict of interest.

## Supplementary material

Supplementary file 1 Key resources that informed the development of initial draft learning outcomes

Supplementary file 2 Feedback from round two

Supplementary file 3 Learning outcomes on completion of the study
