# Supplementary material for: Exploring emerging learning needs: a UK-wide consultation on environmental sustainability learning objectives for medical education
Source: Int J Med Educ. 2015 Dec 24;6:191–200. doi: 10.5116/ijme.5643.62cd (PMC4691188; doi:10.5116/ijme.5643.62cd)
Supplement: Supplementary file 1 — Key resources that informed the development of initial draft learning outcomes [file ijme-6-191-S1.pdf]

Appendix 1

Key resources that informed the development of initial draft learning outcomes

Case studies from the Sustainable Healthcare Education network, Centre for Sustainable Healthcare were accessed in September 2011 from:

- http://sustainablehealthcare.org.uk/sustainable-healthcare-education and used to inform the initial learning outcomes document.

The following papers were used to inform the consultation draft document

- Bodenheimer T, Lorig K, Homan H. Patient Self-management of Chronic Disease in Primary Care. JAMA: The Journal of the American Medical Association. 2002;288(19):2469-75.
- Climate Change Act 2008. [cited 2012 Apr 19]; Available from: http://www.legislation.gov.uk/ukpga/2008/27/part/1
- Connor A, Lillywhite R, Cooke MW. The carbon footprint of a renal service in the United Kingdom. QJM. 2010;103(12):965-75.
- Costello A, Abbas M, Allen A, Ball S, Bell S, Bellamy R, et al. Managing the health effects of climate change: Lancet and University College London Institute for Global Health Commission. Lancet. 2009;373(9676):1693-733.
- Ecosystems and Human Well-Being: Health Synthesis from the Millennium Ecosystem Assessment. World Health Organisation; 2005. Available from: http://www.who.int/globalchange/ecosystems/ecosys.pdf.
- Kim CS, Spahlinger DA, Kin JM, Billi JE. Lean health care: What can hospitals learn from a world-class automaker? Journal of Hospital Medicine. 2006;1(3):191–9.
- Maller C, Townsend M, Pryor A, Brown P, St Leger L. Healthy nature healthy people: “contact with nature” as an upstream health promotion intervention for populations. Health Promot Int. 2006;21(1):45-54.
- NHS England Carbon Footprint (published 2012). NHS Sustainable Development Unit; 2012. Available from: http://www.sdu.nhs.uk/documents/publications/NHS_Carbon_Footprint_Published_2012.pdf.
- Raffle AE. Oil, health, and health care. BMJ. 2010;341:c4596.
- Rockström J, Klum M. The Human Quest: Prospering Within Planetary Boundaries. Princeton University Press; 2012. Available from: http://www.ecologyandsociety.org/vol14/iss2/art32/main.html.
- Royal Society. Climate change: a summary of the science [Internet]. The Royal Society; 2010. Available from: http://royalsociety.org/climate-change-summary-of-science/.
- Schroeder K, Thompson T, Frith K, Pencheon D. Sustainable Healthcare. Wiley-Blackwell; 2012.
