# Supplementary material for: Exploring emerging learning needs: a UK-wide consultation on environmental sustainability learning objectives for medical education
Source: Int J Med Educ. 2015 Dec 24;6:191–200. doi: 10.5116/ijme.5643.62cd (PMC4691188; doi:10.5116/ijme.5643.62cd)
Supplement: Supplementary file 2 — Feedback from round two [file ijme-6-191-S2.pdf]

Appendix 2

Feedback from round two

1. Helping educators and students to engage
    a. Make relevant to patient care – how will this affect the patient in front of me, clinical cases
    b. Highlight evidence of benefits to patients/public
    c. Need local champions
    d. Engage students
    e. Have sustainability as a theme on national conferences
    f. Use peer teaching
    g. Seek funding
2. Teaching ethical aspects of environmental sustainability,
    a. Embed in duties of a doc, Embedding it in the other duties of health professionals: care of patients today and tomorrow (new strapline of NHS England "future generations') – ethics, externalities, doing more good than harm.
    b. Long term view
    c. SOLUTION focused and POSITIVE, not PROBLEMS focussed and NEGATIVE
    d. Good to hear that it is not all about CLIMATE CHANGE
    e. Dr as a citizen domain (in GMCTD)
3. The impacts of long-term healthcare trends on sustainability and related teaching,
    a. Focus on preventive care
    b. Patient autonomy
    c. Link with public health
    d. Integrate with community care
    e. Integrate with social determinants of health
    f. Integrate with communication
    g. Define sustainability; meeting the needs of today without prejudicing the needs of tomorrow (who currently have no choice and voice)
    h. Sustainability needs to be defined. i.e. the biggest wins come from improving lifestyle and public health and that too much of a focus on carbon/ waste reduction side-line this issue
4. Integrating sustainability across the curriculum,
    a. Need for culture change – society and in healthcare
    b. Support the normative goals with interest of individuals and positivity and hedonic goals
    c. Link with an MDT approach
    d. Make it patient centred – listen to patients. There is a patient and public mandate
5. Implementation and teaching delivery
    a. Integration across curriculum; a cross cutting theme
    b. Also need blocks of teaching on this to avoid it being side lined
    c. Taught by physicians / surgeons so that students can recognise that sustainability is vital for good medical practice in all specialities.
    d. Provide supporting materials and train trainers
    e. 'Train the trainers' programme: rather than doing this centrally, why not suggest it as an initiative and see what range of practices arise?
    f. Multi-stakeholder engagement and involvement – GMC, Royal Colleges, Deans etc., Getting GMC, Royal Colleges to
    endorse the PLO's

Assessment

Important/only way to get this recognised
Way of measuring impact of teaching this – will it affect practice? – How will it be measured?
Portfolio better means of assessing than OSCES / one off assessments, because allows assessment to be reflective/continual
