# Supplementary material for: Exploring emerging learning needs: a UK-wide consultation on environmental sustainability learning objectives for medical education
Source: Int J Med Educ. 2015 Dec 24;6:191–200. doi: 10.5116/ijme.5643.62cd (PMC4691188; doi:10.5116/ijme.5643.62cd)
Supplement: Supplementary file 3 — Learning outcomes on completion of the study [file ijme-6-191-S3.pdf]

Appendix 3

Learning outcomes on completion of the study
Introduction

The global community faces major environmental challenges that impact on the health of current and future generations, especially in those areas that are most economically and geographically vulnerable. We can safeguard health by using sustainable approaches that reduce the environmental impact of our actions. Medical education is central to a sustainable future for healthcare and in September 2011 the General Medical Council asked the Sustainable Healthcare Education (SHE) Network to make recommendations on the priority learning outcomes for sustainability, to inform the on-going development of undergraduate and postgraduate medical curricula.

Although sustainability requires some new curricular material, it is primarily a perspective through which to approach existing topics, such as health inequalities and medical leadership. The learning outcomes have been designed to promote critical thinking, and development of the skills necessary to respond to change and uncertainty.

Priority learning outcomes

a. Describe how the environment and human health interact at different levels.
b. Demonstrate the knowledge and skills needed to improve the environmental sustainability of health systems.
c. Discuss how the duty of a doctor to protect and promote health is shaped by the dependence of human health on the local and global environment.

Expanded learning outcomes

This section provides suggestions for more detailed knowledge and skills relevant to each of the priority learning outcomes:

1. Describe how the environment and human health interact at different levels

Doctor as scholar and scientist

a. Outline the dependence of human health on global and local ecological systems, which supply essentials such as air, water and a stable climate.
b. Discuss the contribution of human activity and population size to global environmental changes such as climate change, biodiversity loss and resource depletion.
c. Describe the mechanisms by which human health is affected by environmental change, for example through changes in disease vectors, exposure to extreme weather, migration and reduced food security.
d. Describe features of a health-promoting local environment, in community and healthcare settings, to include access to green spaces, clean air and an active travel infrastructure.

2. Demonstrate the knowledge and skills needed to improve the environmental sustainability of health systems

Doctor as practitioner

a. Define the concept of environmental sustainability.
b. Explain how trends in demographics, technology, and climate and resource availability may affect our ability to provide healthcare into the future.
c. Describe, with examples, the different types of environmental impact resulting from healthcare provision, and how these may be measured.
d. Identify ways to improve the environmental sustainability of health systems - in individual practice, in health service management, and in the design of care systems.
e. Identify potential synergies between policies and practices that promote environmental sustainability and those that promote health.

3. Discuss how the duty of a doctor to protect and promote health is shaped by the dependence of human health on the local and global environment.

Doctor as professional

a. Explain how the health impacts of environmental change are distributed unequally within and between populations and the disparity between those most responsible and those most affected by change.
b. Recognise and articulate personal values concerning environmental sustainability, given the relationship between the environment and the health of current and future generations.
c. Discuss ethical tensions between allocating resources to individual patients and protecting the environment upon which the health of the wider community depends.
d. Demonstrate awareness of organisational sustainability policies and the legal frameworks for reducing carbon emissions.
